# Spatiotemporal expression profiling of proteins in rat sciatic nerve regeneration using reverse phase protein arrays

**DOI:** 10.1186/1477-5956-10-9

**Published:** 2012-02-10

**Authors:** David J Bryan, C Robert Litchfield, Jeffrey V Manchio, Tanya Logvinenko, Antonia H Holway, John Austin, Ian C Summerhayes, Kimberly M Rieger-Christ

**Affiliations:** 1 Tissue Engineering Laboratory, Lahey Clinic Medical Center, Burlington, Massachusetts, USA; 2 Ian C. Summerhayes Cell and Molecular Biology Laboratory, Lahey Clinic Medical Center, Burlington, Massachusetts, USA; 3 Institute for Clinical Research and Health Policy Studies, Tufts Medical Center, Boston, Massachusetts, USA; 4 Aushon BioSystems Inc., Billerica, Massachusetts, USA; 5 Department Surgery, Section of General Surgery, Saint Joseph Mercy Hospital, Ann Arbor, Michigan, USA; 6 Department of Plastic and Reconstructive Surgery, Lahey Clinic Medical Center, Burlington, Massachusetts, USA

**Keywords:** Peripheral nerve regeneration, Reverse phase protein array, Extracellular matrix, Proteomics, Growth factors

## Abstract

**Background:**

Protein expression profiles throughout 28 days of peripheral nerve regeneration were characterized using an established rat sciatic nerve transection injury model. Reverse phase protein microarrays were used to identify the spatial and temporal expression profile of multiple proteins implicated in peripheral nerve regeneration including growth factors, extracellular matrix proteins, and proteins involved in adhesion and migration. This high-throughput approach enabled the simultaneous analysis of 3,360 samples on a nitrocellulose-coated slide.

**Results:**

The extracellular matrix proteins collagen I and III, laminin gamma-1, fibronectin, nidogen and versican displayed an early increase in protein levels in the guide and proximal sections of the regenerating nerve with levels at or above the baseline expression of intact nerve by the end of the 28 day experimental course. The 28 day protein levels were also at or above baseline in the distal segment however an early increase was only noted for laminin, nidogen, and fibronectin. While the level of epidermal growth factor, ciliary neurotrophic factor and fibroblast growth factor-1 and -2 increased throughout the experimental course in the proximal and distal segments, nerve growth factor only increased in the distal segment and fibroblast growth factor-1 and -2 and nerve growth factor were the only proteins in that group to show an early increase in the guide contents. As expected, several proteins involved in cell adhesion and motility; namely focal adhesion kinase, N-cadherin and β-catenin increased earlier in the proximal and distal segments than in the guide contents reflecting the relatively acellular matrix of the early regenerate.

**Conclusions:**

In this study we identified changes in expression of multiple proteins over time linked to regeneration of the rat sciatic nerve both demonstrating the utility of reverse phase protein arrays in nerve regeneration research and revealing a detailed, composite spatiotemporal expression profile of peripheral nerve regeneration.

## Background

Peripheral nerve injury has been estimated to occur in about 3% of trauma patients and can lead to life-long disability [1]. Transection injuries, especially those that result in large gaps between nerve ends, are particularly incapacitating. While the peripheral nervous system has the ability to regenerate, transection injuries typically require reconstructive surgery and the restoration of sufficient function remains a significant challenge.

Regeneration in the peripheral nervous system is a complex process which requires the careful orchestration of multiple factors and cues to create the optimal microenvironment for regeneration to occur [reviewed in [2] and [3]]. Past research has shown that axons regenerate from the proximal stump of a transected nerve in response to tactile signals and chemotropic secretions from the distal stump of the severed nerve [4,5]. The cellular events underlying serial stages in peripheral nerve regeneration have been described and include Wallerian degeneration of the distal nerve followed by regeneration events led by the migration of Schwann cells from the distal nerve segment and axon sprouting from the proximal nerve segment forming the proximal growth cone [reviewed in [6] and [7]]. These axons use bands of Büngner, formed by proliferating Schwann cells extending from the distal stump, as a physical scaffold to guide their growth. Recently, Parrinello and colleagues reported that fibroblasts also play a key role in peripheral nerve regeneration. More specifically, they have shown that when the nerve is severed, ephrin-B/EphB2 signaling between fibroblasts and Schwann cells results in cell sorting, followed by directional collective cell migration of Schwann cells out of the nerve stumps to guide regrowing axons cross the wound [8]. The axons and Schwann cells both respond to and produce, trophic factors which are transported to the injury site where they have growth potentiating effects; attracting axons in a concentration guided, modality-specific and organ-specific manner [9-12]. The role of Schwann cells following axotomy is multifaceted including initial phagocytosis of cell debris followed by the transfer of degraded myelin to macrophages, as part of the degenerative process. The migration of Schwann cells precedes the involvement of alternative cell populations activated by signals presumably released by the cells of the proximal growth cone that in turn respond to triggers occurring physically downstream. Such cues for migration include growth factors and extracellular matrix (ECM) proteins as has been demonstrated in *in vitro *models of migration [13-15].

The underlying events that drive the regenerative process have yet to be fully elucidated; although there has been extensive research looking at both inhibitory and promotional factors. Peripheral nerve regeneration research efforts can be generally divided into two categories, those that evaluate materials used to create a physical scaffold or nerve guide to direct the regenerating nerve (also referred to as tubulation) [16] and those that alter the environment of the regenerating nerve. Environmental alteration, for example through the use of cultured cells or growth factors, is often combined with a guide, to create an optimal regenerative environment [reviewed in [17] and [18]]. While early studies used conduits composed of decalcified bone, more recent guides have consisted of a range of synthetic and biological materials [reviewed in [17] and [18]]. The ideal guide material is one that retains sufficient mechanical strength for surgical manipulation while providing permeability and bioresorbability so as not to act as a barrier for cell infiltration and nerve regeneration [[19-24] and others]. While many studies have shown promising results, they have yet to produce an alternative that is superior to the nerve autograft, the current gold standard. Although autografting is the standard of care when repairing loss of length of large multifascicular nerve injuries, it is not without disadvantages, namely neuroma formation, unavailability of donor nerve, conflicts of modality and donor site morbidity [25-28].

Research focused on altering the microenvironment of the regenerating nerve has also shown promise. A number of molecules have been shown to enhance nerve regeneration including nerve growth factor (NGF) [29-32], fibroblast growth factor (FGF) [33-37], glial growth factor [38], platelet-derived growth factor [39], ciliary neurotrophic factor (CNTF) [40-43] and ECM components such as fibronectin, collagen and laminin [44-49]. The action of each class of molecule can be direct, influencing axon growth by binding to axonal cellular components or indirect by guiding axon growth and modeling a microenvironment permissive for the promotion of regeneration. Despite the advances reported in experimental models which have combined the different facets of polymer guides and the delivery of relevant molecules directly to the regenerative site, full functional restoration has not been attained. The events underlying peripheral nerve regeneration represent tightly orchestrated interactions between a diverse array of molecules within a tightly regulated temporal sequence. Thus it is difficult to adopt a reductionist approach to understand such a complex series of events.

To further enhance our understanding of the regenerative process it would be beneficial to gain a more global view of the cellular and biochemical events that underlie peripheral nerve regeneration from both a spatial and temporal perspective by evaluating protein expression in different segments of the regenerating nerve over time. Proteomic techniques such as Western blots and immunohistochemistry haven been used successfully for spatiotemporal profiling, but these techniques are typically low-throughput and most reports focus on just a few proteins. Due to differences in experimental models, it is difficult to combine observations between studies. Proteomics techniques such as two-dimensional gel electrophoresis and mass spectrometry have been instrumental in identifying candidate proteins involved in peripheral nerve regeneration and have demonstrated the complexity of protein expression in the regenerating nerve [50-53]. The reverse phase protein array (RPPA) is a powerful proteomic technique that enables the measurement of protein levels in a large number of samples simultaneously [54-60]. By arraying lysates created from distinct portions of the regenerating nerve at distinct time points we were able to create a spatiotemporal expression profile of the peripheral nerve regeneration process on a single microscope slide. Each slide requires only a small amount of lysate (~2 nl per spot), so the material from a single experiment can be used to evaluate the expression profile of hundreds of proteins.

In this study we evaluate the use of RPPAs in peripheral nerve regeneration using an established rat sciatic nerve transection injury model combined with a polyethylene nerve guide. The non-permeable, non-degradable polyethylene guide was selected for this study over a resorbable guide to simplify the harvesting of regenerated tissue. Previous studies by our group using this model have reproducibly resulted in regenerated nerve cables within 4-8 weeks [61] and the series of events leading to regeneration in a similar silicone guide model are well described [62]. The expression of 15 proteins known to be involved in various aspects of the regenerative process including growth factors, ECM proteins, and adhesion and motility proteins was profiled in multiple locations and at multiple time points.

## Results

### Validation of antibodies for use in RPPA

**Figure 1:**
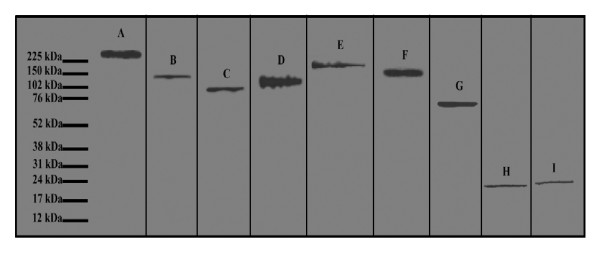
**Antibody Validation**: Validation of selected antibodies in western blot analysis showing detection of a single band for fibronectin (**A**), N-cadherin (**B**), β-catenin (**C**), E-cadherin (**D**), laminin γ-1 (**E**), nidogen (**F**), versican (**G**), FGF-2 (**H**), and CNTF (**I**).

All antibodies were tested for their suitability for use in RPPA by western blot analysis (Figure 1 and data not shown). Total protein lysates from excised nerves were pooled and separated by gel electrophoresis. Confirmation of the utility of each antibody for use in the RPPA setting was determined by the detection of a single predominant band by western blot. An example blot with a representative selection of the antibodies used is shown in Figure 1.

### Reverse phase protein arrays

**Figure 2:**
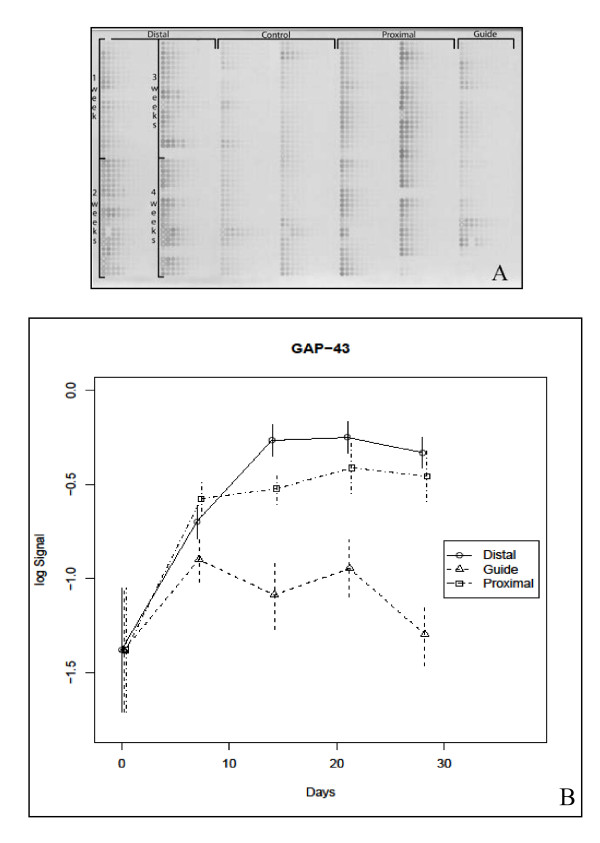
**GAP-43 RPPA**: (**A**) RPPA probed with GAP-43 antibody showing an increase in protein expression over a 28-day regenerative period. (**B**) A graphic representation of the microarray showing the expression levels of GAP-43 over time in each segment.

The proximal and distal ends of the severed sciatic nerve, along with the solid contents of the guide, were harvested at different time points following axotomy and tissue was prepared for RPPA. Tissue lysates were arrayed in a 10-point, 2-fold dilution series on a single nitrocellulose-coated microscope slide, with tissue controls corresponding to the day 0 excised sciatic nerve from that animal. All samples from all animals used in this study were printed on every slide. The expression level of 15 different proteins was measured in the tissue lysate preparations. Figure 2 shows the processed RPPA for growth associated protein 43 (GAP-43) (A) as well as the extrapolated protein level as a function of time for each of the regions (proximal, guide and distal) (B).

### Expression profile of the selected protein panel throughout nerve regeneration

**Figure 3:**
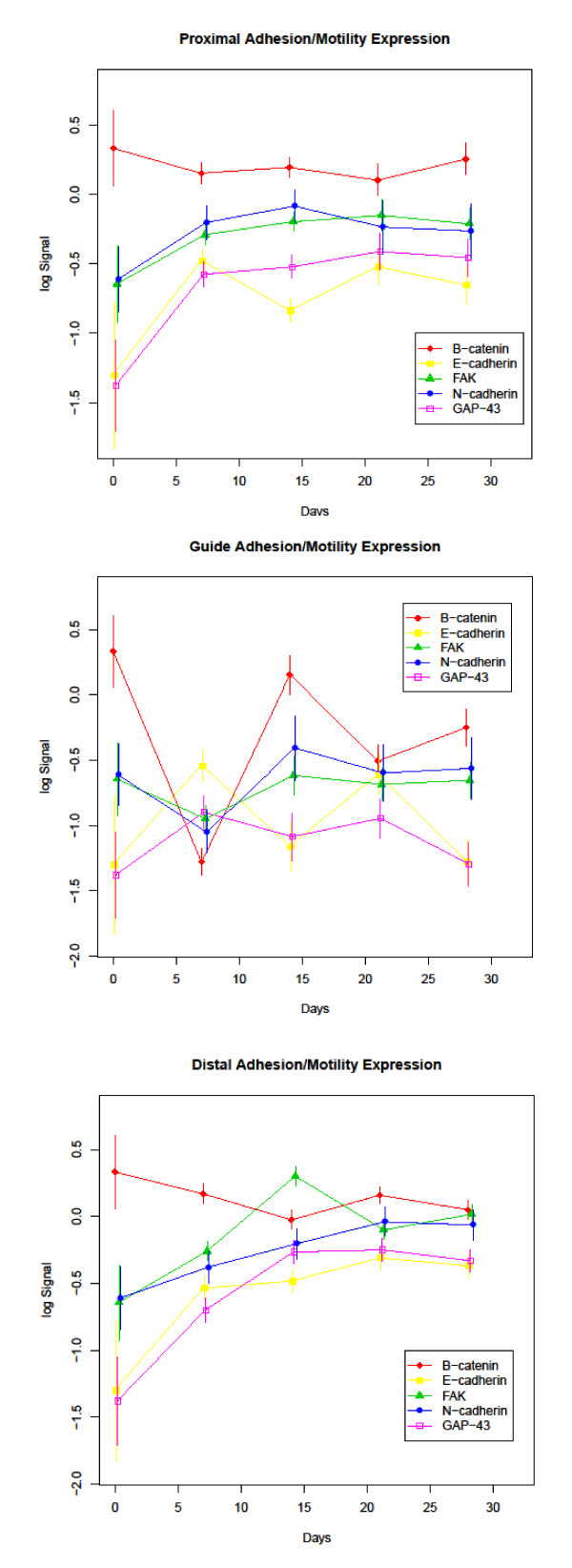
**Adhesion and Motility Protein Expression**: Expression profiles of the adhesion and motility group of study proteins divided into proximal, guide and distal segments. Time (in days) is plotted on the X-axis and log normalized data on the Y axis.

**Figure 4:**
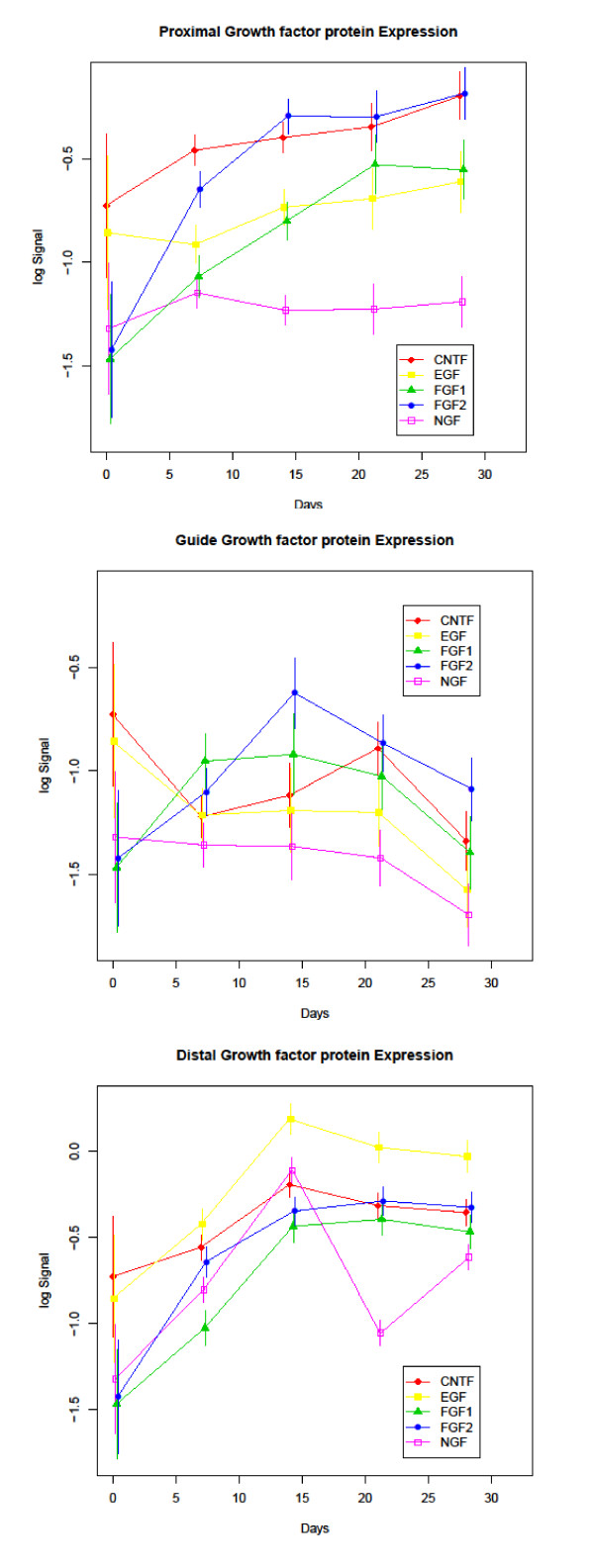
**Growth Factor Protein Expression**: Expression profiles of the growth factor group of study proteins divided into proximal, guide and distal segments. Time (in days) is plotted on the X-axis and log normalized data on the Y axis.

**Figure 5:**
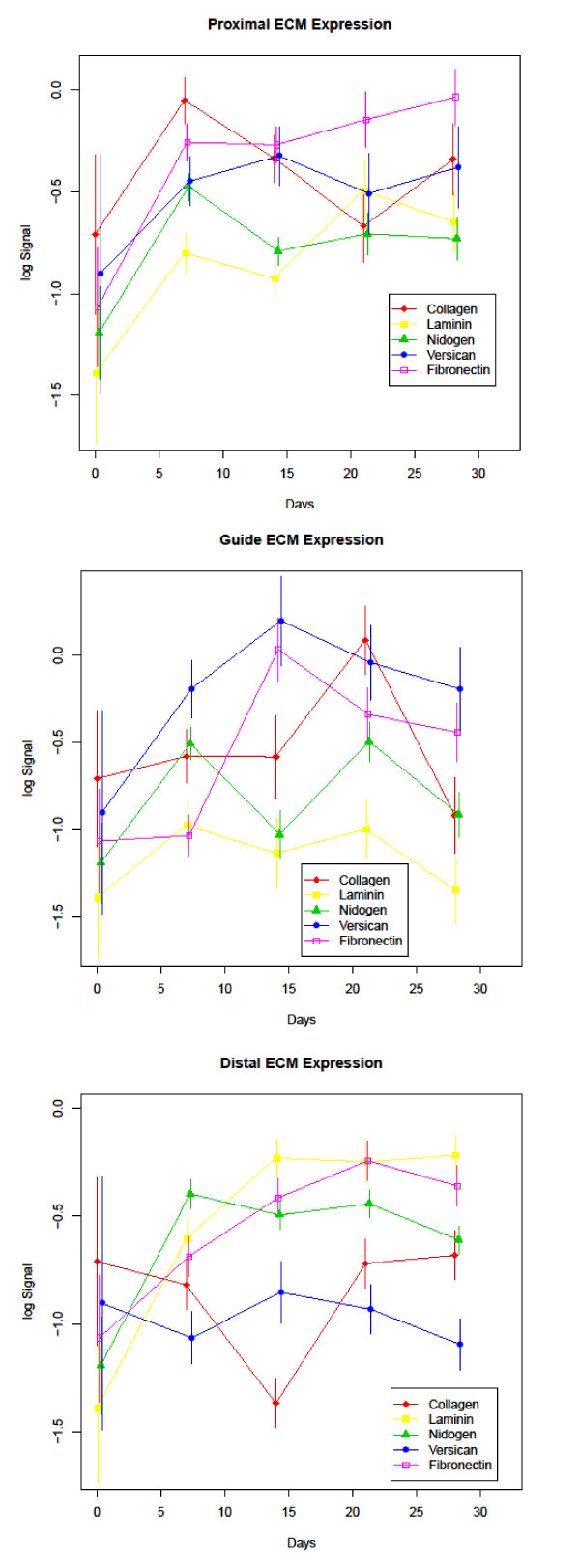
**ECM Protein Expression**: Expression profiles of the ECM group of study proteins divided into proximal, guide and distal segments. Time (in days) is plotted on the X-axis and log normalized data on the Y axis.

**Figure 6:**
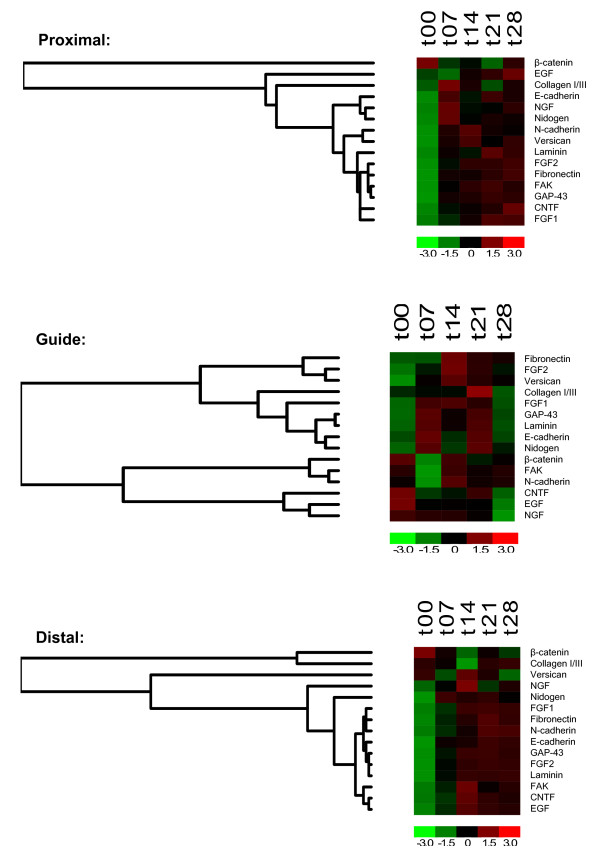
**Hierarchical Clustering Analysis in Heatmap Format**: Proximal (**A**), distal (**B**), and solid guide content (**C**) samples are shown for each protein over time points of ≤ 7, 14, 21, and 28 days. Colors corresponding to relative levels of expression are shown at the bottom of each heatmap.

Protein expression profiles throughout the 28 day experimental period are displayed in Figures 3, 4, and 5. The antibody panel selected for this study is divided into three groups. The first group is adhesion and cell motility consisting of E- and N-cadherin, β-catenin, and focal adhesion kinase (FAK) (Figure 3). The growth factors NGF, FGF-1, FGF-2, epidermal growth factor (EGF) and CNTF comprise the second group (Figure 4). ECM proteins laminin, fibronectin, collagen I/III, nidogen and versican make up the third group (Figure 5). While GAP-43 plays direct roles in different aspects of nerve regeneration with functional effects spanning more than one of these groups, it is grouped with the adhesion and motility proteins. A summary of expression of the complete panel of proteins under study is shown in Figure 6 presented in a heatmap format.

### Adhesion/motility associated proteins

Members of the classic cadherin family, E- and N-cadherin, displayed an overall increase in expression throughout the course of regeneration. In the proximal and distal segments of the regenerating nerve this increase was significant for E-cadherin when the expression level from day 0 (intact nerve) was compared with day 28 (q-values of 0.022 and 0.002 respectively). The change in N-cadherin expression between day 0 and day 28 was significant in the distal portion only (q-value 0.036). Interestingly, the levels of N-cadherin in the proximal segment appeared to peak at day 14 and the expression level at 14 days was significantly higher than baseline (q-value 0.024). Both E-cadherin and N-cadherin displayed continued elevated expression up to 21 days in the distal segment followed by a plateau in expression up to 28 days. There was no significant change in E- or N-cadherin levels in the guide contents when comparing the day 0 to the day 28 time point. The guide segment results compare intact nerve (day 0 expression levels) to the regenerate which, at least for the early time points, contains no nerve. Since the guide segment contains only regenerated tissue, the lack of significant difference from day 0 represents a return to the baseline protein levels seen in intact nerve. For N-cadherin this return to baseline occurs by day 14 while fluctuations in E-cadherin expression were recorded throughout the 28 day regenerative period with above baseline peaks at ≤ 7 and 21 days and troughs that approached baseline levels at 14 and 28 days. In contrast, β-catenin, an integral member of the cadherin complex, showed a significant change from baseline only at day ≤ 7 in the proximal (q-value 0.024) and distal (q-value 0.029) segments, but in the guide tissue displayed peaks and troughs that mirrored N-cadherin, but were opposite to that recorded for E-cadherin in the same site.

FAK has been shown to promote neurite outgrowth and in this model displayed an increase in expression throughout the regenerative process in the proximal and distal segments with an initial, significant drop at day ≤ 7 (q-value 0.002) and recovery to higher than baseline levels by day 14 in the guide portion. The overall increase in FAK levels between day 0 and day 28 was significant in the distal segment only (q-value 0.016).

In the distal nerve stump GAP-43 levels increased rapidly up to 14 days followed by a relative plateau for the remainder of the experimental course. A sharp increase in GAP-43 was also seen in the proximal nerve up until 7 days with a slower, but marked increase through 28 days. In contrast, GAP-43 protein level showed no significant change from baseline throughout the course of the experiment in the guide contents indicating an early up-regulation of GAP-43 in the regenerating tissue. The overall increase in GAP-43 between day 0 and day 28 was significant in the proximal and distal segments only (q-values 0.002 and 0.001 respectively).

### Growth factors

Amongst the molecules included in the growth factor group, a significant increase in the expression level of FGF-1, FGF-2 and EGF was recorded between 0-14 days in the regenerating distal nerve followed by a plateau in expression between 14 and 28 days (the q-value for the day 0 to day 14 comparisons were 0.007, 0.004, and 0.009 respectively). In the proximal nerve segment, expression levels of each of the aforementioned growth factors continued to rise past 14 days although none of the changes after day 14 reached significance. Comparing day 0 to day 28, both FGF-1 and FGF-2 demonstrated a significant increase in protein levels in the proximal nerve segment, but EGF did not (q-values for FGF-1 and FGF-2 were 0.006 and < 0.001 respectively). Interestingly, significantly reduced EGF expression was recorded in the guide tissue over the 28 day regeneration period (q-value < 0.001). In contrast, a comparison of day 0 to day 28 protein levels for FGF-1 and FGF-2 did not reveal a significant change in the guide contents indicating a return to the baseline levels of intact nerve and both proteins displayed a temporary increase in expression that peaked at 14 days and reached significance for FGF-2 (q-value 0.037).

CNTF displayed only a limited, insignificant increase in expression in the proximal and distal segments relative to the other growth factors in the study and, like EGF, significantly reduced expression in the guide tissue between day 0 and day 28 (q-value < 0.001). However, unlike EGF, CNTF did not decrease throughout the entire regenerative period, instead displaying an increase in expression at 21 days followed by a further decline at 28 days.

NGF displayed a biphasic expression profile in the regenerating distal nerve with significant peaks occurring at 14 and 28 days post-axotomy (q values < 0.001 and 0.006 respectively when comparing day 14 and day 28 to baseline). No significant change in NGF expression was recorded in the proximal segment or guide compartment of the regenerating nerve between baseline and day 28. In the guide compartment the level of NGF was not significantly different than baseline by day ≤ 7 indicating an early recovery of NGF protein in this segment.

### Extracellular matrix proteins

The ECM proteins have been shown to play a role in growth promotion and cell migration involving directional guidance, both important processes in nerve regeneration. Fibronectin displayed a significant increase in protein levels over baseline at 21 and 28 days in the proximal segment (q-values 0.014 and 0.024 respectively), 21 days in the distal segment (q-value 0.036), and even at the earliest time point of ≤ 7 days no significant change from intact nerve in the guide segment. A significant increase from baseline levels was observed in the guide segment at day 14 and day 21 (q-values < 0.001 and 0.023 respectively). Laminin displayed a significant increase in both the proximal and distal segments between 0 and 28 days (q values 0.009 and < 0.001 respectively), whereas the guide contents did not show a significant change when comparing the same time points indicating a return to the levels of intact nerve. Like fibronectin, day ≤ 7 laminin expression was also not significantly different than baseline in the guide segment. Collagen I/III had a distinct temporal sequence of expression which peaked at days ≤ 7 and 21 in the proximal and guide segments, respectively, and was significantly reduced compared to baseline in the distal segment (q-value 0.011) at 14 days followed by restoration to expression levels recorded in the intact nerve for the remainder of the experimental course. At day ≤ 7 the guide segment had recovered to baseline collagen I/III expression levels. Nidogen and versican protein levels were elevated overall although the change was only significant in the distal segment (q-values for both comparisons were 0.014). Nidogen, a potentiator of Schwann cell proliferation, showed biphasic expression with peaks at ≤ 7 and 21 days post-axotomy most notably in the guide segment indicating an early increase even higher than baseline expression. Versican protein levels were elevated early in the proximal and guide segments with a significant increase at 14 days (q-values 0.011 and ≤ 0.001 respectively) followed by a slow decline with levels increased, but not significantly different than baseline at 28 days. In the distal segment protein levels were significantly lower than baseline at day ≤7 and day 28 (q-values 0.018 and 0.014 respectively).

## Discussion

Significant advances in promoting peripheral nerve regeneration have been made on multiple fronts including the use of bioresorbable polymer guides to serve as conduits and delivery devices for different molecules that have been implicated as promotional in the regenerative process. Knowledge of the expression pattern of proteins essential to regeneration have helped predict the best candidates for exogenous administration, however this information must be pooled from individual studies with variable models in order to generate a global expression profile. In this study we used RPPA to facilitate the simultaneous temporal and spatial expression mapping of key proteins in the nerve regeneration process using a rat sciatic nerve transection injury model. The benefit of RPPA analysis is that many samples can be analyzed simultaneously. Since this technique requires very little starting material, it also enables the analysis of a large number of proteins. The limited number of proteins reported in this study was not due to a limitation in source material, but instead the limited availability of antibodies that cross-react with rat proteins and were suitable for use in RPPA. In this study every antibody was first validated by western blot and any antibody that did not produce a single predominant band by western was excluded. Although an analysis of 15 proteins does not represent the full capability of the RPPA technique, we believe that this paper provides significant insight into the regulation of these proteins and sets the stage for further proteomic evaluation using this model.

The phases of regeneration in a non-permeable, non-resorbable nerve guide chamber have been described and include a fluid, matrix, cellular, and axonal phase [62]. Within a day after axotomy and guide insertion the guide fills with fluid that contains neuronotrophic factors. A mainly acellular matrix forms within a week followed by the immigration of cells (including Schwann, fibroblast, and endothelial) from both the proximal and distal nerve stumps after 7-14 days. Finally, after 2 weeks, axonal elongation begins with myelination occurring a few days later [62]. According to this description, the first time point used in the current study (≤ 7 days) would capture the regenerative process at the matrix phase with the second time point (14 days) representing the cellular phase. The final time points of 21 and 28 days represent the axonal phase with the later time point likely to have a higher percentage of myelinated axons.

Protein expression analysis in the guide content compares intact nerve (day 0 expression levels) to the regenerate which initially contains no nerve. Based on the described regenerative process, an early increase (manifested as a return to baseline levels) in the proteins included in the ECM category would be expected in the guide portion of the regenerate. Indeed, all of the ECM proteins studied demonstrate not only a return to baseline, but an increase in expression at ≤ 7 days over intact nerve. In contrast, several of the cellular proteins such as β-catenin, FAK, and N-cadherin show an initial decline from the protein level of intact nerve in the guide segment and peak later at day 14 corresponding with the cellular phase of regeneration. This initial decline from baseline is to be expected since the guide segment does not contain intact nerve at the earlier time points. Interestingly, the growth factors represent a mixed group in the guide portion with an early return to baseline or increase over intact nerve for NGF, FGF-1 and FGF-2, and overall decline in the levels of CNTF and EGF in the solid guide contents. Overall, we saw a decrease in the expression of all growth factors in the guide contents from day 21 to day 28, the time at which axonal migration and myelination is underway. This is consistent with a previous study that demonstrated that the neuronotrophic activity of the fluid that fills the guide chamber is highest in the first few days of regeneration suggesting that the role of at least some of these molecules peaks early in the regenerative process [63]. The general patterns of protein expression seen by RPPA analysis in this study are consistent with established knowledge and warrant more thorough analysis.

GAP-43 is present in the presynaptic terminal of novel neuromuscular junctions [64], and has been shown to be expressed in regenerating axons following nerve injury [65-68]. GAP-43 reportedly plays a promotional role in nerve sprouting post axotomy [69]. In our model we found an overall increase in GAP-43 levels throughout the experiment. Several groups have reported an early up-regulation of GAP-43 protein or mRNA following nerve injury [68,70,71]. We observed a pattern of early, increased expression in both the proximal and distal nerve segments that appeared to plateau by 14 days. At its peak, the distal nerve segment has a higher expression level of GAP-43 than the proximal nerve and the guide contents show no significant change in GAP-43 compared to intact nerve even at the earliest time point. These results differ slightly than those of Plantinga et al. who reported no increase in proximal GAP-43 mRNA levels, but this difference might be explained by differences in the experimental models, most notably that the nerve ends in the former study were coagulated and ligated while ours were allowed to regenerate. The discrepancy could also be explained by poor correlation between mRNA and protein levels [68].

Members of the cadherin family and the associated proteins that form the functional cadherin/catenin complex have been shown to play important roles in cell migration. E-cadherin is associated with the adherens junctions of the Schmidt-Lanterman incisures and is unique because it forms an autotypic adherens-type junction confined to the plasma membrane of a single Schwann cell [72]. Expression of E-cadherin is increased following injury in a variety of models [73,74]. Consistent with these studies, we found elevated expression of E-cadherin in all segments at the day ≤ 7 time point. In the distal and proximal segments, day 28 levels remain significantly higher than baseline while in the guide they return to the baseline level of intact nerve.

As cadherin complexes assemble, there is a distinct hierarchy of catenin association led by E-cadherin/β-catenin binding followed by additional catenin members. Interestingly, β-catenin that binds to E-cadherin within the cadherin complex, revealed no significant change in expression profile throughout the regenerative process in the proximal and distal segments in our study. β-catenin levels in the guide decreased significantly at ≤ 7 days followed by a rebound to baseline levels and a significant but lower decline from baseline at 21 and 28 days. Our observation is in contrast to a report by Hatoko et al. that showed increased β -catenin expression in the graft portion of a nerve graft model of regeneration, however this difference might be explained by the fact that the previous study was evaluating expression in intact nerve grafts where our study included just the regenerated contents within a hollow guide [75].

N-cadherin has been shown to be present on axonal growth cones *in vitro *[76] and is thought to play a role in the stabilization of myelin sheaths based on its distribution and low expression levels in normal nerves [77,78]. Several groups have reported increased N-cadherin levels at 15 days post-axotomy [79,80]. We confirmed an increase in N-cadherin expression during regeneration that reached statistical significance at 14 days in the proximal segment and 21 days in the distal segment, but did not observe significantly higher expression in the distal segment than the proximal segment as reported by Thorton and colleagues [80]. This discrepancy may be explained by differences in the models used where in the Thorton study the nerve ends were capped following axotomy and not allowed to regenerate. We also showed that in the distal stump, N-cadherin expression peaks at 21 days which is later than the 14 day peak in the proximal segment and suggests a temporal sequence of events. The guide contents show an initial (day ≤ 7) decrease in N-cadherin expression followed by a higher than baseline peak at 14 days and slight decline similar to the pattern observed in the proximal segment. Since N-cadherin has been shown to be expressed around regenerated axons [79,81], the drop in N-cadherin expression in the guide contents at day ≤ 7 is likely due to the absence of axon-axon or axon-Schwann cell interactions at this location at such an early stage in the regenerative process.

FAK has been identified as a regulator of Schwann cell proliferation in the developing nervous system [82] and inhibition of FAK activity inhibited neurite growth in an *in vitro *model [83]. In the current study, FAK expression was increased in both the distal and proximal nerve segments although only the increase in the distal portion reached statistical significance. In the guide there was a drop in FAK levels at day ≤ 7, likely due to the absence of intact nerve and corresponding reduction in Schwann cells at this time point, followed by a return to baseline for the remainder of the experiment. Activation of FAK leads to autophosphorylation at Tyr397 and an analysis of activated FAK levels will be needed to further elucidate the role of FAK in regeneration.

Numerous growth factors have been implicated in peripheral nerve regeneration with different members of the FGF family playing a prominent role in this process [reviewed in [84] and [85]]. Several groups have reported enhancement of nerve regeneration using FGF-1 and -2 [33,34,86-89] and FGF-2 levels have shown to be up-regulated following peripheral nerve injury at both the mRNA and protein levels [[90-92] and others]. Surprisingly, a decrease in FGF-1 protein levels or mitogenic activity after crush or transection has also been reported [93,94]. We observed an increase in FGF-1 and FGF-2 levels in both the proximal and distal ends of the regenerating nerve. Interestingly, in our study, FGF-1, FGF-2, and NGF were the only growth factors to show an early increase in the guide contents suggesting that these factors play a role in the initial phase of the regenerative process.

While EGFR mRNA and protein levels increase following nerve injury [95], and an EGF homolog has been shown to promote axonal regeneration in CNS neurons *in vitro *[96], Dubuisson et al. found that exogenous EGF did not enhance peripheral nerve regeneration in their model [97]. We found that EGF protein levels were increased significantly in the distal segment. The proximal segment showed an insignificant increase and there was an overall decrease in EGF in the solid contents of the guide. This differential in EGF expression could imply a role for EGF in the maturation of peripheral nerve tissue rather than in the more active state of regeneration.

NGF is a neurotrophin that has been shown to play a role in nerve regeneration in a large number of studies. Rich et al. demonstrated improved peripheral nerve regeneration with added NGF, as have others [30-32,98]. NGF mRNA and protein has been shown to be expressed at low levels in healthy nerves and up-regulated in the distal, but not the proximal stump upon injury [94,99]. In our model NGF expression remained at baseline levels in the regenerating proximal segment. In contrast, NGF expression rapidly returned to the baseline level of intact nerve in the guide tissue and was significantly elevated in the distal segment by 14 days post-axotomy and at the end of the experiment following an unexplained dip at 21 days. This supports the earlier findings that NGF is linked to events in the guide and distal segment of the regenerating nerve.

CNTF increases neuronal survival, neurite outgrowth, and axonal regeneration with exogenous administration [41,42,100,101], but Ito and colleagues reported low CNTF mRNA levels post-axotomy and a similar observation has been made in a crush model [102,103]. In our study CNTF displayed only a limited, insignificant increase in expression in the proximal and distal segments and an overall significant decrease in protein levels within the solid guide contents consistent with these later reports.

ECM components play an essential role in nervous system development and repair. Schwann cells assemble a fibrillar network that consists of fibronectin, laminin, and collagen type IV which is thought to play a role in proliferation [104]. The different ECM proteins likely exert their influence on different facets of nerve regeneration influencing multiple cellular processes. Fibronectin has previously been shown to be a potent chemoattractant for Schwann cell migration *in vitro *and *in vivo *[13,14,105,106] and fibronectin protein and mRNA levels were found to be elevated in several models of nerve injury [107-109]. We found an increase in fibronectin levels that reached significance at day 21 proximally and distally. Within the guide there was an early return to baseline levels by day ≤ 7 and significant increase over baseline by day 14. Fibronectin levels increased more rapidly in the proximal segment than the distal segment and were significantly higher at ≤ 7 days in the proximal segment consistent with the findings of Lefcort et al. who demonstrated highest fibronectin expression in the vicinity of the injury at the same time point [107].

Laminins are key components of the basal lamina and laminin has been shown to play a prominent promotional role in nerve regeneration *in vivo *by regulating axonal growth [110,111]. The laminin gamma-1 (B2) chain is one of the most abundant of the 12 known chains that compose the laminin heterotrimer and is present in laminin isoforms 1-4 and 6-11 [112]. There is conflicting evidence regarding gamma-1 mRNA expression with Wallquist and colleagues showing an up-regulation after sciatic transection (most notably in the proximal stump) and Doyu et al. reporting a decrease when normalized to total RNA levels [113,114]. In our experiment, gamma-1 protein expression increased significantly between day 0 and day 28 both proximally and distally, and there was an early return to baseline gamma-1 levels in the guide itself. Our results are most consistent with those of Wallquist [113]. In both our model and the Wallquist model the nerve ends were allowed to regenerate, while in the Doyu studies they were not [113,114]. This difference in experimental design may explain the conflicting results.

Collagen is the major component of the ECM and several groups have shown an increase in collagen I and III mRNA after nerve injury as well as functional improvement with exogenous administration [108,115-118]. In the current study, we used an antibody that detected both collagens I and III and found no significant change in levels between day 0 and day 28 in the proximal and distal segments. In the guide there was an early return to baseline protein levels by day ≤ 7 followed by a significantly higher than baseline expression level at day 21 and a significantly lower level than baseline level by day 28 suggesting that, at least at the protein level, the role of collagens I/III is predominantly in the regenerating portion of the nerve.

Nidogen is a component of the basement membrane. Nidogen protein and mRNA levels have been shown to be up-regulated post transection injury [15] and nidogen appears to be required for neurite outgrowth after axotomy [119]. Our results support these findings as we found an increase in nidogen levels early in the regenerative process with a significant increase by ≤ 7 days in all segments. The guide contents displayed a biphasic increase in the guide with significant peaks at ≤ 7 and 21 days and baseline levels at the 14 and 28 days. Clearly nidogen plays a role in many aspects of the regenerative process.

The chondroitin sulphate proteoglycan, versican, isoform V2 has been shown to be an inhibitor of axonal growth while V1 has been shown to promote neurite outgrowth [120-122]. We looked at the versican V0/V1 isoforms and found a significant increase in the proximal and guide segments that peaked at 14 days followed by a slight decline to the end of the study period consistent with these reports. Interestingly, versican levels remained low throughout the experiment in the distal segment suggesting that versican plays more of a role in the proximal region of the regenerating nerve during the timeframe we analyzed.

Not surprisingly, cluster analysis of protein expression over time did not always separate the proteins into the functional groups we defined, but other interesting patterns were revealed (Figure 6). In the proximal section, increased expression of N-cadherin was recorded throughout the regenerative period consistent with its reported role in cell migration and expression in cells of mesenchymal origin. In embryoid bodies, the timing and spatial distribution of versican expression correlates with the appearance and localization of N-cadherin [123]. In this study, N-cadherin and versican expression clustered together in the proximal segment of the regenerating nerve, suggesting that the regenerative events in the proximal nerve may mimic the processes that occur in early development.

Analysis of expression levels in the guide content is particularly interesting as this compares intact nerve (day 0 expression levels) to the regenerate which, at least for the early time points, contains no nerve. It is not surprising that a greater number of proteins show an initial decline in this segment, but interestingly many are up-regulated even at the earliest time point. In the guide segment groupings more closely follow functional roles. For example, all of the ECM proteins are represented in a single branch (early up-regulated expression) and the majority of the adhesion and motility proteins in the other (early down-regulated expression). The pattern of expression correlates to the temporal role of these proteins groups in the regenerative process where ECM proteins play an early role in the matrix phase and adhesion and motility proteins a later role in the cellular phase of regeneration. GAP-43 is clustered in the up-regulated branch and grouped closely with laminin. A functional relationship between GAP-43 and laminin has been implicated in several other studies and this expression pattern may reflect the complex role of GAP-43 in the regenerative process [124,125].

## Conclusions

The expression of multiple cellular and extracellular molecules is finely orchestrated and integrated leading to the regeneration of a new nerve. Past work using this or similar models of peripheral nerve regeneration have taken a reductionist approach evaluating the contribution of individual or small numbers of proteins in the regenerative process. The RPPA approach taken in this study allows expression profiling of multiple proteins from a large number of samples simultaneously. In this study we focused on proteins known to be involved in the regenerative process and with known expression profiles. The results both validated previous observations and provided new detail and complexity, but the application can be easily expanded to include any protein with a suitable, validated antibody including posttranslational modifications such as phosphorylation. In this study we have demonstrated that RPPA is a reliable, high-throughput approach for the analysis of overall protein expression profiles with the potential to identify the temporal sequence of events underlying complex, multifaceted, biological processes.

## Methods

### Surgical procedure

All animal experiments were performed with the approval of the Institutional Animal Care and Use Committee at Lahey Clinic. Forty male Sprague Dawley rats weighing between 225-250 g served as the model for nerve guide repair. All animals were anesthetized via an intraperitoneal injection of 45 mg kg^-1 ^sodium pentobarbital. All underwent an initial surgery during which the right sciatic nerve was exposed via a three centimeter incision made just posterior to the femoral head. The dissection was carried down through the gluteal muscle at which point the sciatic nerve was identified. The remainder of the dissection was performed under an operating microscope at 30 × magnification. This same approach was carried out during a second harvesting procedure.

A length of at least 20 mm of nerve was exposed. A 10 mm section was removed via sharp transection with straight micro-scissors leaving at least a 5 mm proximal and distal sciatic nerve stump. A 14 mm long polyethylene nerve guide (1.67 mm ID, Becton Dickinson) was sutured in place with 10-0 nylon suture, using a horizontal mattress stitch. The suture was placed 2 mm from the end of the guide and through the epineurium on each respective nerve stump. The full circumference of each stump was therefore drawn entirely inside the guide with 2 mm of length lying inside each end of the guide, resulting in the proximal and distal stumps positioned 10 mm apart. The 10 mm resected section of nerve was divided into two 5 mm sections (5 mm to act as a per-animal control, and 5 mm to be used for antibody validation) and the two sections were placed in individual tubes, and frozen immediately in liquid nitrogen. The gluteal muscle was then closed with three buried interrupted 3-0 chromic sutures and the skin was closed with a running 4-0 braided polyglycolic acid suture.

At each time point (≤ 7, 14, 21, and 28 days), 10 rats underwent a second procedure to harvest the proximal and distal nerve ends and the contents of the guide. All adhesions on the guide were carefully taken down with sharp dissection under the operating microscope. Sharp transection of the proximal and distal stump was carried out, 5 mm from each end of the guide, with straight micro-scissors. The entire specimen, proximal and distal stumps within the guide, was taken out as one piece. The 10-0 nylon sutures were cut and the remaining tissue was separated into proximal and distal nerve stumps and solid guide contents. Proximal and distal segments included a total of 7 mm of nerve (2 mm of stump within the guide and 5 mm of additional nerve). At each time point of ≤ 7, 14, 21, and 28 days there were 5, 2, 6, and 4 rats with solid material spanning the guide, respectively (see Table 1 for experimental overview). Solid guide content was defined as any matrix or tissue that could be macroscopically retrieved from the lumen of the nerve guide. The guide content segment was comprised of only regenerated material and not the nerve stumps themselves. Only tissue from animals with solid guide content was included in the analysis. All tissue was frozen immediately in liquid nitrogen.

**Table 1:** Experimental Overview

	Number of Animals Included in Day 0 (Baseline) Measurements	Number of Rats Harvested at End Point	Number of Rats with Solid Guide Contents at End Point
**Day ≤ 7**	40	10	5
		
**Day 14**		10	2
		
**Day 21**		10	6
		
**Day 28**		10	4

At each time point proximal, distal and solid guide contents were harvested from each animal. Only tissue from animals with solid guide content was included in the analysis

### Antibody validation

Nerve lysate for antibody validation was prepared by crushing a pooled sample of the excised normal 5 mm nerve segments under liquid nitrogen, using a mortar and pestle. The finely ground tissue was lysed in hot sample buffer (2 × ESB - 0.08 M Tris, pH 6.8; 0.07 M SDS, 10% glycerol, 0.001% bromophenol blue and 1 mM CaCl_2_) and sheared through a 26-gauge needle. β -mercaptoethanol (1%) was added to each sample which was boiled for 5 min. The resulting lysate was loaded across the top of a 7.5% polyacrylamide resolving gel, with one lane containing a marker. Proteins were transferred overnight onto nitrocellulose. Membranes were blocked in 10% milk in TBS with 0.05% Tween-20, cut into strips and placed on primary antibody overnight at 4°C. Blots were washed in TBS with 0.05% Tween-20, three times for 15 min each, and secondary antibody linked to horseradish peroxidase was incubated with the blots for 60 min at room temperature. Blots were then washed as described above and developed with an ECL kit (Amersham, Arlington Heights, IL). Only antibodies that produced a single band by western blot analysis were used for RPPA.

### Antibodies

Validated antibodies used were: NGF (Epitomics, Burlingame, CA); Nidogen and FGF1 (Santa Cruz Biotechnology, Santa Cruz, CA); CNTF and FAK (Millipore, Billerica, MA); FGF2, Fibronectin, GAP-43, E-cadherin and β-catenin (BD Biosciences, San Jose, CA) EGF and laminin-1 gamma-1 (R&D Systems, Minneapolis, MN); Collagen Type I/III (Calbiochem, San Diego, CA); Versican (Sigma-Aldrich, St. Louis, MO); and N-cadherin (Zymed, San Francisco, CA). The manufacturer of the fifteen antibodies as well as the concentrations used in RPPA and western blot are shown in Table 2.

**Table 2:** Antibody Name, Vendor, and Dilutions Used for Western Blotting and RPPA Applications

Antibody Name	Vendor	Dilution
NGF-b	Epitomics	1:500

Nidogen-1 (H-200)	SCBT	1:500

CNTF (4-68)	Millipore	1:100

FGF2 (basic)	BD Biosciences	1:500

FGF1 (acidic)	SCBT	1:100

EGF	R & D Systems	1:250

Laminin-1 gamma1	R & D Systems	1:1000

Collagen type I/III	Calbiochem	1:300

Fibronectin	BD Biosciences	1:5000

GAP-43	BD Biosciences	1:1000

FAK	Millipore	1:500

Versican V0/V1	Sigma-Aldrich	1:1000

E-cadherin	BD Biosciences	1:500

N-cadherin	Zymed	1:500

β-catenin	BD Biosciences	1:1000

### Preparation of tissue lysates

Each nerve sample was crushed under liquid nitrogen with a clean mortar and pestle and suspended in 1.0 ml ice-cold PBS and centrifuged at 4000 rpm for 2 min. After removing the PBS, a volume of lysis buffer, composed of 9 M Urea, 4% Chaps, 2% Pharmalyte pH 8.0-10.5 and 65 mM Dithiothreitol, equal to the size of the pellet, was added and aspirated up and down 3 times. Samples were centrifuged at 14,000 rpm for 30 min. The supernatant was collected and stored at -80°C and the pellet was discarded. All steps following the initial PBS wash were performed at 4°C. Ten, 2-fold serial dilutions were made from each lysate with a buffer containing 6 M urea, 2.7% CHAPS, 1.3% Pharmalyte (pH 8.0-10.5), and 43.6 mM Dithiothreitol [126,127].

### Reverse-phase protein arrays

Samples were arrayed using the Aushon 2470 solid pin, contact microarrayer equipped with 185 μm pins (Aushon BioSystems, Billerica, MA). The print matrix was a nitrocellulose-cast glass slide (Grace BioLabs, Bend, OR). Each slide contained 10, 2-fold serial dilutions of each sample and included a technical replicate totaling 3,360 lysate spots per slide. All printing was carried out at 80% relative humidity.

### Developing

Printed nitrocellulose-coated slides were washed for two 15-min periods in deionized water. Slides were blocked for 1 h in I-Block (Life Technololgies-0.2% I-Block, 0.01% Tween-20 in phosphate buffered saline) with continuous shaking at room temperature. Immunostaining of microarrays was performed on an automated stainer (Autostainer Plus, Dako, Carpinteria, CA) using a tyramide-based catalyzed signal amplification system according to the manufacturer's protocol (K1500, Dako, Carpinteria, CA). A negative control was included using a non-specific antibody solution (Dako, Carpinteria, CA) substituting for the primary antibody.

### Image processing & quantification

The slides were scanned at 2400 dpi resolution in an uncompressed, 16-bit TIFF image format using an optical flatbed scanner. Images were processed by the P-SCAN and ProteinScan (version 0.21) programs written using Matlab software (MathWorks, Natick, MA) by the National Institutes of Health (NIH campus, Bethesda, MD). Outliers traceable to defects in the arrays were eliminated and the data were analyzed. The numerical output for each sample was generated by a modified Dose Interpolation algorithm, DI_25 _[128]. ProteinScan is a program package that automates the modified DI algorithm. The DI_25 _value of each nerve sample was normalized for total protein concentration determined by colloidal gold staining.

### Statistical analysis

The protein intensity data was log-transformed to ensure data normality. To investigate the spatial and temporal differences in the protein expression levels, a linear mixed effects model was applied to each protein separately. The models contained fixed effects for different times and sample types and are adjusted for baseline protein expression levels. To account for correlated data structure random rat effect was used in the models. To determine which particular times and samples were significantly different, Scheffe's test [129] was used. The significance results were presented as q-values, false discovery rate computed based on p-values to adjust for multiple testing [[130,131], see Additional file 1-q values]. All statistical analyses were performed using R version 2.9.1 [132] and R packages nlme [133] and q-value [134]. The clustering and heatmaps were produced using dChip software [135-137]. Heatmap data was row-standardized to have a mean of 0.

## Abbreviations

ECM: Extracellular matrix; NGF: Nerve growth factor; FGF: Fibroblast growth factor; CNTF: Ciliary neurotrophic factor; RPPA: Reverse phase protein array; GAP-43: Growth associated protein 43; FAK: Focal adhesion kinase; EGF: Epidermal growth factor

## Competing interests

AHH and JA were employees of Aushon BioSystems (manufacturer of the 2470 microarrayer) at the time that this study took place. AHH's affiliation at the time of publication is with Lahey Clinic.

## Authors' contributions

CRL and JVM performed animal surgeries. CRL performed antibody validation experiments, reverse phase microarray processing and assisted with manuscript preparation. DJB contributed to study design, data interpretation, and critical manuscript review. TL performed statistical analysis. AHH contributed to study design and data interpretation, performed reverse phase microarray printing and drafted the manuscript. JA-performed reverse phase microarray printing and critical review of the manuscript. ICS contributed to study design and data interpretation. KMR-C contributed to study design, data interpretation, and critical review of the manuscript. All authors read and approved the final manuscript.

## Supplementary Material

Additional file 1 **q-values; The statistical significance of expression data was determined and results are presented as q values, false discovery rate adjusted p-values**. Click here for file
